# Sitagliptin‐mediated preservation of endothelial progenitor cell function *via* augmenting autophagy enhances ischaemic angiogenesis in diabetes

**DOI:** 10.1111/jcmm.13296

**Published:** 2017-08-10

**Authors:** Xiaozhen Dai, Jun Zeng, Xiaoqing Yan, Qian Lin, Kai Wang, Jing Chen, Feixia Shen, Xuemei Gu, Yuehui Wang, Jun Chen, Kejian Pan, Lu Cai, Kupper A. Wintergerst, Yi Tan

**Affiliations:** ^1^ Chinese‐American Research Institute for Diabetic Complications School of Pharmaceutical Sciences School of Nursing at the Wenzhou Medical University Wenzhou China; ^2^ School of Biomedicine Chengdu Medical College Chengdu China; ^3^ Pediatric Research Institute Department of Pediatrics University of Louisville School of Medicine Louisville USA; ^4^ Department of Medical Genetics and Cell Biology School of Basic Medical Sciences Guangzhou Medical University Guangzhou China; ^5^ Department of Pharmacology and Toxicology University of Louisville School of Medicine Louisville USA; ^6^ Departments of Pediatrics, Endocrinology and Metabolism the First Affiliated Hospital of Wenzhou Medical University Wenzhou China; ^7^ Departments of Geriatrics the First Hospital of Jilin University Changchun China; ^8^ Department of Pediatrics Division of Endocrinology Wendy L. Novak Diabetes Care Center University of Louisville Louisville KY USA

**Keywords:** sitagliptin, angiogenesis, hind limb ischaemia, endothelial progenitor cells, autophagy

## Abstract

Recently, the dipeptidyl peptidase‐4 (DPP‐4) inhibitor sitagliptin, a major anti‐hyperglycaemic agent, has received substantial attention as a therapeutic target for cardiovascular diseases *via* enhancing the number of circulating endothelial progenitor cells (EPCs). However, the direct effects of sitagliptin on EPC function remain elusive. In this study, we evaluated the proangiogenic effects of sitagliptin on a diabetic hind limb ischaemia (HLI) model *in vivo* and on EPC culture *in vitro*. Treatment of *db/db* mice with sitagliptin (Januvia) after HLI surgery efficiently enhanced ischaemic angiogenesis and blood perfusion, which was accompanied by significant increases in circulating EPC numbers. EPCs derived from the bone marrow of normal mice were treated with high glucose to mimic diabetic hyperglycaemia. We found that high glucose treatment induced EPC apoptosis and tube formation impairment, which were significantly prevented by sitagliptin pretreatment. A mechanistic study found that high glucose treatment of EPCs induced dramatic increases in oxidative stress and apoptosis; pretreatment of EPCs with sitagliptin significantly attenuated high glucose‐induced apoptosis, tube formation impairment and oxidative stress. Furthermore, we found that sitagliptin restored the basal autophagy of EPCs that was impaired by high glucose *via* activating the AMP‐activated protein kinase/unc‐51‐like autophagy activating kinase 1 signalling pathway, although an autophagy inhibitor abolished the protective effects of sitagliptin on EPCs. Altogether, the results indicate that sitagliptin‐induced preservation of EPC angiogenic function results in an improvement of diabetic ischaemia angiogenesis and blood perfusion, which are most likely mediated by sitagliptin‐induced prevention of EPC apoptosis *via* augmenting autophagy.

## Introduction

Diabetes is a disease that is strongly associated with both microvascular and macrovascular complications, including retinopathy, nephropathy, and neuropathy (microvascular) and ischaemic heart disease, peripheral vascular disease and cerebrovascular disease (macrovascular), resulting in organ and tissue damage in approximately one‐third to one‐half of people with diabetes 1, 2. Vascular complications associated with diabetes are the leading causes of morbidity and mortality for diabetic patients. Vascular complications in diabetes are associated with dysregulation of vascular remodelling and vascular growth, decreased responsiveness to ischaemic/hypoxic stimuli, impaired or abnormal neovascularization, and a lack of endothelial regeneration. Thus, there is a great need for therapeutic interventions aimed at accelerating the repair of dysfunctional endothelium and restoring blood flow in damaged organs and tissues 3.

Endothelial progenitor cells (EPCs) are found in bone marrow, peripheral blood and certain organs, such as the spleen and liver. In the event of endothelial injury or tissue ischaemia, EPCs are mobilized into the circulation from the bone marrow, home to the site of injury, differentiate into mature endothelial cells and incorporate into the endothelium, replacing apoptotic or damaged cells and mediating neovascularization. However, the number of EPCs in diabetic patients is reduced, and the function of EPCs is attenuated as a consequence of exposure to the dysmetabolic diabetic environment 4. An efficient therapeutic strategy includes mobilizing more EPCs into peripheral blood and promoting EPCs homing to ischaemic tissue to enhance angiogenesis with pharmacological agents.

Dipeptidyl peptidase 4 (DPP‐4), a membrane‐bound extracellular peptidase, also designated CD26, has been demonstrated to cleave cytokines and chemokines 5. Stromal‐derived factor‐1α (SDF‐1α), one of the chemokine substrates of DPP‐4[5], serves as a chemoattractant for EPCs and stem/progenitor cells and plays critical roles in EPCs and stem cell mobilization and homing 6. Previous studies have reported that inhibition of DPP‐4 activity in the blood leads to an increase in the circulating number of EPCs 7, 8, 9, enhancing angiogenesis and blood flow in hind limb ischaemia (HLI) 8, 9 and protecting the heart in models of myocardial infarction 10, 11. However, the direct effects of DPP‐4 inhibition on EPCs function remain elusive.

In this study, sitagliptin, a DPP‐4 inhibitor that is widely used in the clinic, was used to treat HLI in *db/db* type 2 diabetic mice *in vivo* and EPCs under high glucose (HG) conditions *in vitro*. We aimed to evaluate (1) the therapeutic effects of sitagliptin on HLI; (2) the mobilization of EPCs induced by sitagliptin; (3) and the proangiogenic effects and mechanisms of sitagliptin on EPCs under HG conditions, which was used to mimic a dysmetabolic environment.

## Materials and methods

### Animals

*db/db* (FVB background) male mice at 2–3 months of age were used in this study. All animal procedures were approved by the Animal Policy and Welfare Committee of Wenzhou Medical University and/or the Institutional Animal Care and Use Committee of the University of Louisville, which conform to the Guide for the Care and Use of Laboratory Animals published by the US National Institutes of Health (publication No. 85‐23, revised 1996).

### HLI and sitagliptin treatment

The *db/+* founder mice [FVB.BKS(D)‐Lepr^*db/+*^/ChuaJ, FVB background] were purchased from the Jackson Laboratory (Bar Harbor, ME, USA) and maintained under specific pathogen‐free conditions at the University of Louisville Animal Facility (Louisville, KY, USA) or the Wenzhou Medical University. The *db/db* mice were generated by breeding male *db/+* to female *db/+* mice following Jackson Laboratory's instructions. Twelve‐week‐old male *db/db* mice were equally divided into two groups (*n* = 16/group) to develop the HLI model as described in our previous report 12. One group was pretreated with sitagliptin (25 mg/kg, daily) for 1 week before HLI surgery and continually treated with sitagliptin for an additional 7 or 35 days after surgery *via* gavage administration. Another group was treated with H_2_O as the vehicle control. At 7 or 35 days after surgery, eight mice from each group were sacrificed to collect blood and gastrocnemius muscle samples. The HLI procedure is briefly described below: under sufficient anaesthesia with isoflurane (1–3% isoflurane in 100% oxygen at a flow rate of 1 l/min.), the hind limbs were shaved and the entire right superficial femoral artery and vein (from just below the deep femoral arteries to the popliteal artery and vein) were ligated with 6‐0 silk sutures, cut and excised with an electrical coagulator (Fine Science Tools Inc., Foster City, CA, USA). The overlying skin was closed with 4‐0 silk sutures.

### Measurement of blood flow perfusion with a Pericam perfusion speckle imager (PSI)

To evaluate the limb perfusion ratio [ischaemic limb (right)/normal limb (left)], real‐time microcirculation imaging analysis was performed using a Pericam PSI based on laser speckle contrast analysis technology (Perimed Inc., Kings Park, NY, USA) before surgery and at day 0, 3, 7 14, 21, 28 and 35 post‐surgery.

### Quantification of circulating EPCs by flow cytometry

At days 3 and 7 after surgery, blood samples were collected in 0.1 mol/l EDTA‐2Na‐coated tubes from the tail vein. Whole blood was incubated with CD34‐PE and VEGFR‐2‐APC antibodies 13, 14 (BD Pharmingen, San Jose, CA, USA) for 1 hr, then lysed and fixed in FACS™ lysing solution (BD Biosciences, San Jose, CA, USA) for 5 min. Flow cytometry analysis was performed with a BD FACS (BD Biosciences) to count VEGFR‐2 and CD34 double‐positive EPCs. Isotype control IgG (BD Pharmingen) was used to exclude false‐positive cells.

### Determination of SDF‐1 and glucagon‐like peptide‐1 (GLP‐1) in plasma

At day 14 after sitagliptin administration (7 days after surgery), the plasma was collected to detect the levels of SDF‐1 and GLP‐1. The levels of SDF‐1α and GLP‐1 were measured with commercially available enzyme‐linked immunosorbent assay (ELISA) kits CXCL12/SDF‐1α immunoassay (R&D systems, Minneapolis, MN, USA) and Glucagon Like Peptide‐1 (Active) ELISA kit (Millipore, Billerica, MA, USA), respectively.

### Histological assessment

The extent of angiogenesis at day 35 post‐ischaemic surgery was assessed by measuring capillary density using isolectin B4 staining. Ischaemic gastrocnemius muscle tissues were fixed with 4% paraformaldehyde and embedded with paraffin. Paraffin sections (5 μm) were stained with Alexa Fluor^®^ 594 conjugated isolectin GS‐IB4 antibody (Thermo Scientific, Waltham, MA, USA) to evaluate the capillary density. The capillaries were counted in randomly selected fields for a total of 20 different fields (×40 magnification) per section and three sections per animal. The capillary density is presented as capillary number per muscle fibre.

### Isolation and culture of bone marrow‐derived EPCs

EPCs were isolated from the bone marrow of wild‐type (WT) FVB mice and cultured according to our established methods with minor modifications 15. Briefly, bone marrow mononuclear cells (MNCs) were isolated from the femurs and tibias of the mice by density gradient centrifugation with histopaque‐1083 (Sigma‐Aldrich, St. Louis, MO, USA). After two washes, MNCs were plated on vitronectin‐coated culture dishes (Sigma‐Aldrich) and maintained in endothelial growth factor‐supplemented media (EGM‐2 bullet kit; Lonza, Basel, Switzerland) with 10% foetal bovine serum. Cells were cultured at 37°C with 5% CO_2_ in a humidified atmosphere. EGM‐2 medium was replaced after the first 24 hrs and every 3 days thereafter. Cell colonies appeared after 7 days of culture and were expanded to the fourth or fifth passage for further analysis.

### Characterization of bone marrow‐derived MNCs

After 14 days of culture in endothelial‐specific media and the removal of non‐adherent MNCs, the remaining cells were characterized by uptake of Dil‐Ac‐LDL and ulex europaeus lectin‐1 binding. For the uptake of Dil‐Ac‐LDL and ulex europaeus lectin‐1 binding assay, cells were seeded on a vitronectin‐coated 8‐well μ‐slide (ibidi, Martinsried, Germany) one day before the procedure, and then, the cells were incubated with 5 μg/ml acetylated DiI lipoprotein from human plasma (Dil‐Ac‐LDL, Thermo Fisher Scientific, Waltham, MA, USA) at 37°C for 4 hrs, followed by three washes with Dulbecco's phosphate‐buffered saline (DPBS) and fixed with 4% paraformaldehyde. Then, the cells were incubated with 10 μg/ml fluorescein isothiocyanate‐labelled ulex europaeus lectin‐1 (FITC‐UEA‐1; Sigma‐Aldrich) for 1 hr at room temperature. After incubation, the cells were rinsed with DPBS three times and were visualized *via* confocal microscopy.

To further confirm the identity of these cells, immunofluorescence staining was performed to analyse the expression of specific cell markers VEGFR2 and Sca‐1. For the immunofluorescence staining, cells were seeded on a vitronectin‐coated 8‐well μ‐slide one day before the procedure and then fixed with 4% paraformaldehyde after three washes with DPBS. The fixed cells were incubated with PE‐VEGR2 and FITC‐Sca‐1 16, 17 antibodies (Abcam, Cambridge, MA, USA) overnight at 4°C. DAPI (4′,6‐diamidino‐2‐phenylindole, dihydrochloride) was used to label nuclei.

### DPP‐4 enzymatic activity assay

DPP‐4 enzymatic activity was assayed in the culture medium of EPCs under different treatment conditions using a DPP‐4 Activity Assay Kit (Sigma‐Aldrich). EPCs were seeded on 12‐well plates and maintained under basal culture conditions with or without HG (25 mmol/l) for 24 hrs 18 in the presence or absence of different concentrations of sitagliptin. The culture medium was collected for the DPP‐4 enzymatic activity assay. Briefly, a 50 μl volume of medium was diluted with 48 μl of DPP‐4 assay buffer, mixed with 2 μl substrate gly‐pro‐7‐amino‐4‐methylcoumarin (AMC) and then incubated at 37°C for 30 min. The release of AMC from the substrate was measured with a fluorescence spectrophotometer at 360 nm excitation and 460 nm emission.

### Apoptosis assay

EPCs were seeded on 12‐well plates (1 × 10^5^ cells/well) and maintained under basal culture conditions or with HG (25 mmol/l) for 24 hrs in the presence or absence of sitagliptin. Non‐adherent cells were removed by washing with PBS. In some experiments, cells were pretreated with the autophagy inhibitor 3‐methyladenine (3‐MA, 5 mmol/l; Sigma‐Aldrich) or the autophagy activator rapamycin (10 μmol/l; Sigma‐Aldrich) for 30 min. and then continually exposed to HG for the indicated durations in the presence or absence of sitagliptin. Subsequently, adherent cells were released with 0.25% trypsin without EDTA. EPCs were collected by centrifugation and stained with an APC‐conjugated Annexin V Apoptosis Detection Kit according to the manufacturer's instructions (Biolegend, San Diego, CA, USA). Apoptotic EPCs were detected by flow cytometry. Early apoptotic cells were defined as AnnexinV^+^/PI^–^.

### Tube formation assay

The *in vitro* angiogenic capability of EPCs was determined by a Matrigel tube formation assay. Briefly, 48‐well plates were coated with growth factor‐reduced matrix gel (150 μl/well, BD Biosciences). EPCs (5 × 10^4^ cells/well) in 200‐μl basal culture medium or medium containing HG in the presence or absence of different concentrations of sitagliptin were incubated at 37°C with 5% CO_2_ for 12 hrs to form tubes. In some experiments, cells were pretreated with 3‐MA (5 mmol/l) or rapamycin (10 μmol/l) for 30 min. and then continually exposed to HG for the indicated durations in the presence or absence of sitagliptin. Images of tubes in each well were acquired using an inverted microscope (Nikon Eclipse E600, Nikon, Kanagawa, Japan). The tube lengths were calculated by ImageJ software (National Institutes of Health, Bethesda, MD, USA).

### Quantitative determination of oxidative stress

To detect the reactive oxygen species (ROS) levels of EPCs with HG treatment in the presence or absence of different concentrations of sitagliptin, a dihydroethidium (DHE; Molecular Probes, Eugene, OR, USA) probe was used to stain EPCs. DHE is cell permeable and able to react with superoxide to form ethidium, which in turn intercalates with DNA and produces nuclear fluorescence. EPCs were seeded on 24‐well plates, treated with HG in the presence or absence of sitagliptin for 6 hrs and then incubated with 5 μmol/l DHE in PBS for 30 min. at 37°C. Nuclear DHE‐positive staining indicates superoxide generation in cells. Fluorescence intensity was detected by a microplate reader (SpectraMax M3; Molecular Devices, Sunnyvale, CA, USA) under specific wave length conditions (excitation = 518 nm; fluorescence = 605 nm). Meanwhile, fluorescence images of random EPCs were captured at 20× magnification (XI 71 Olympus, Tokyo, Japan).

### Autophagic signalling assay

Autophagic signalling was assayed by detecting microtubule‐associated protein 1A/1B‐light chain 3 (LC3) and p62 protein expression levels and LC3‐bound puncta formation. For the LC3 and p62 protein expression assay, EPCs were seeded on 6‐well plates (1 × 10^5^ cells/well) and maintained under basal culture conditions with or without HG for 24 hrs in the presence or absence of sitagliptin. Non‐adherent cells were removed by washing with PBS. In some experiments, cells were pretreated with AMP‐activated protein kinase (AMPK) inhibitor compound C (10 μmol/l; Sigma‐Aldrich) for 30 min. and then continually exposed to HG for the indicated durations in the presence or absence of sitagliptin. Then, EPCs were harvested for a Western blot assay of LC3 and p62 protein expression levels. For the LC3‐bound puncta assay, EPCs were seeded on an 8‐well microculture chamber (2 × 10^4^ cells/well) and maintained under basal culture conditions with or without HG for 24 hrs in the presence or absence of sitagliptin. Non‐adherent cells were removed by washing with PBS. The remaining cells were stained with a LC3B primary antibody (1:100; Novus Biologicals, Littleton, CO, USA) and an appropriate FITC‐conjugated secondary antibody. LC3 puncta were observed with immunofluorescence microscopy.

### Western blot assay

Western blotting was performed as described in a previous study 15. Gastrocnemius muscle tissues and harvested cells were homogenized or lysed in ice‐cold RIPA lysis buffer (Santa Cruz Biotechnology, Dallas, TX, USA). The protein concentration was determined using a Bradford protein assay kit (Bio‐Rad, Hercules, CA, USA). The total proteins were separated by 10% sodium dodecyl sulphate polyacrylamide gel electrophoresis (SDS‐PAGE) and transferred to nitrocellulose membranes (Bio‐Rad). The membranes were blocked in tris‐buffered saline with 5% non‐fat milk and 0.5% bovine serum albumin for 1 hr and then incubated with primary antibody overnight at 4°C, followed by an incubation with secondary antibodies for 1 hr at room temperature after standard washing procedures. The primary antibodies against VEGF (1:10,000), glyceraldehyde 3‐phosphate dehydrogenase (GAPDH, 1:5000) and β‐actin (1:3000) were purchased from Santa Cruz Biotechnology; SDF‐1 (1:2000), AMPK and p‐AMPK (1:2000), mTOR and p‐mTOR (1:2000), unc‐51‐like autophagy activating kinase 1 (ULK1) and p‐ULK1 and P62 (1:2000) were purchased from Cell Signaling Technology (Danvers, MA, USA); and LCB (1:2000) was purchased from Novus Biologicals (Littleton, CO, USA). All horseradish peroxidase (HRP)‐conjugated secondary antibodies were purchased from Santa Cruz Biotechnology. Blots were visualized with SuperSignal West Femto Maximum Sensitivity Substrate (Thermo Scientific) and quantified with Quantity 5.2 software (Bio‐Rad).

### Statistical analysis

All data are presented as the mean ± S.D. Statistical analysis was performed using Origin 7.5 (OriginLab data analysis and graphing software, Northampton, MA, USA) with one‐way or two‐way anova, followed by *post hoc* multiple comparisons with Scheffe's test. Statistical significance was considered *P* < 0.05.

## Results

### Sitagliptin enhances blood perfusion and angiogenesis in HLI in type 2 diabetic mice

**Figure 1:**
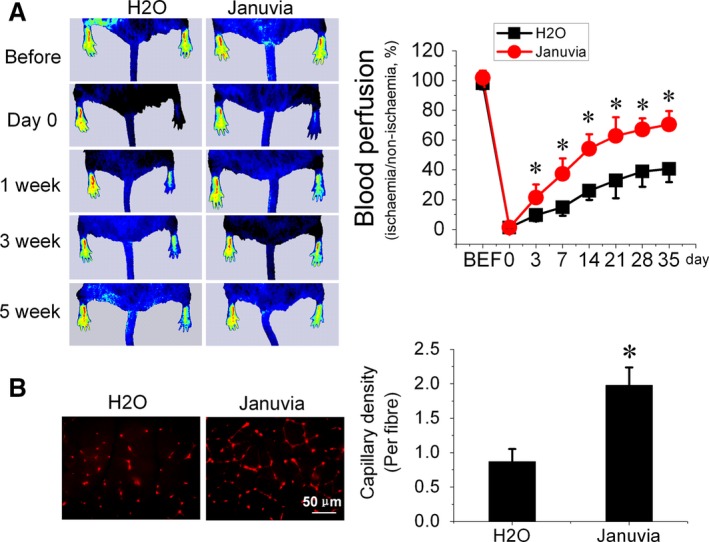
Sitagliptin enhances blood flow recovery and angiogenesis in hind limb ischaemia (HLI) in type 2 diabetic mice. The proangiogenic effects of sitagliptin were investigated in HLI in *db/db* type 2 diabetic mice. (**A**) The time course of blood perfusion and quantitative analysis before and after HLI surgery with or without Januvia (sitagliptin, 25 mg/kg body weight, daily) treatment. Blood perfusion was calculated as the ratio of ischaemic to non‐ischaemic limb perfusion measured by a Pericam perfusion speckle imager (PSI). (**B**) Immunofluorescent staining and quantification of isolectin‐positive capillaries in transverse sections of gastrocnemius muscle tissue from ischaemic hind limbs 35 days after HLI surgery. Capillary density is expressed as isolectin‐positive capillaries per muscle fibre. *n* = 8 mice per group. Data shown in graphs represent the means ± S.D. *P < 0.05 vs H_2_O group.

To evaluate the angiogenic effect of DPP‐4 inhibition in diabetes, *db/db* mice were pretreated with sitagliptin (Januvia) for 1 week; then, the mice were subjected to HLI surgery and continually treated with sitagliptin for an additional 5 weeks until to the end of the experiments. Blood flow was evaluated by PSI at day 3, 7, 14, 21, 28 and 35 after surgery. The results showed that sitagliptin treatment time dependently improved blood perfusion in *db/db* mice underwent HLI (Fig. 1A), which was accompanied by promoted angiogenesis mirrored by increased capillary density (Fig. 1B).

### Sitagliptin increases proangiogenic factor expression levels and enhances EPCs mobilization in type 2 diabetic mice

**Figure 2:**
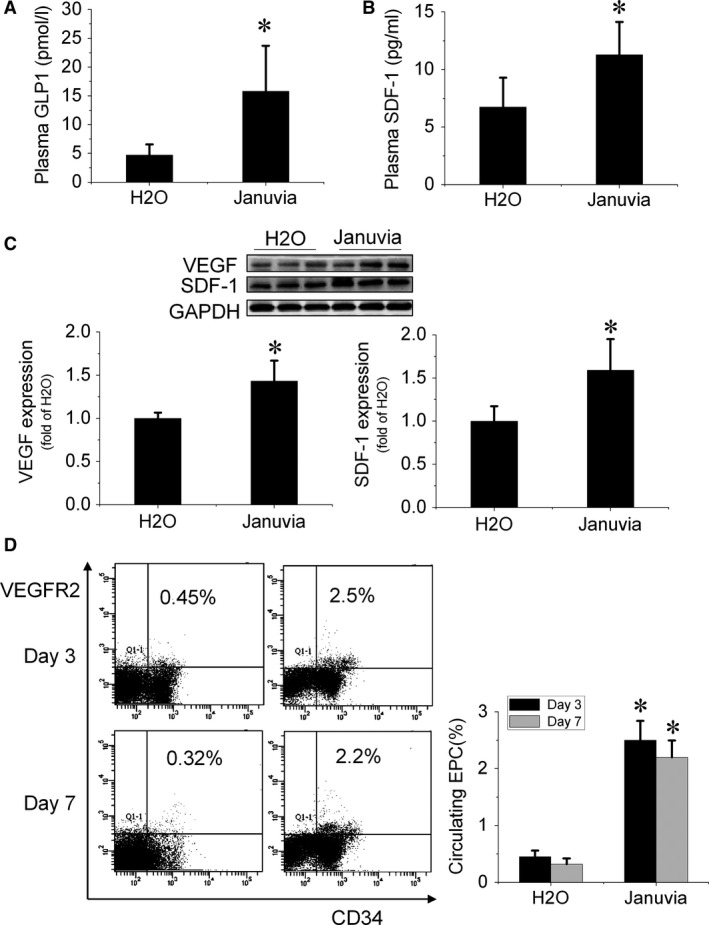
Sitagliptin increases proangiogenic factor expression and enhances endothelial progenitor cells (EPCs) mobilization in type 2 diabetic mice. (**A**,** B**) The expression levels of glucagon‐like peptide‐1 (GLP‐1) and stromal cell‐derived factor‐1 (SDF‐1) in plasma after surgery for 7 days were detected by enzyme‐linked immunosorbent assay; (**C**) The expression levels of VEGF and SDF‐1 in gastrocnemius muscle were detected by Western blotting; (**D**) At days 3 and 7 after surgery, peripheral blood was collected to evaluate the number of EPCs (CD34 + /VEGFR2 +) in circulation by a flow cytometry assay. *n* = 8 mice per group. Data shown in graphs represent the means ± S.D. *P < 0.05 vs H_2_O group.

To evaluate the effect of DPP‐4 inhibition on the expression levels of proangiogenic factors in plasma and ischaemic tissue, peripheral blood and gastrocnemius muscles in ischaemic limbs were collected at day 7 after surgery. The ELISA results showed that mice that received sitagliptin had elevated levels of GLP‐1 and SDF‐1 in plasma (Fig. 2A and B). Proangiogenic factors in gastrocnemius muscle were detected by Western blotting, and the results showed that VEGF and SDF‐1 increased significantly in sitagliptin‐treated mice (Fig. 2C).

At days 3 and 7 after surgery, peripheral blood was collected to evaluate the number of EPCs in circulation. A flow cytometry assay showed that the number of circulating EPCs (CD34+/VEGFR2+) significantly increased in sitagliptin‐treated mice (Fig. 2D), indicating that sitagliptin promoted EPCs mobilization from bone marrow to peripheral blood.

### Characterization of bone marrow‐derived EPCs

**Figure 3:**
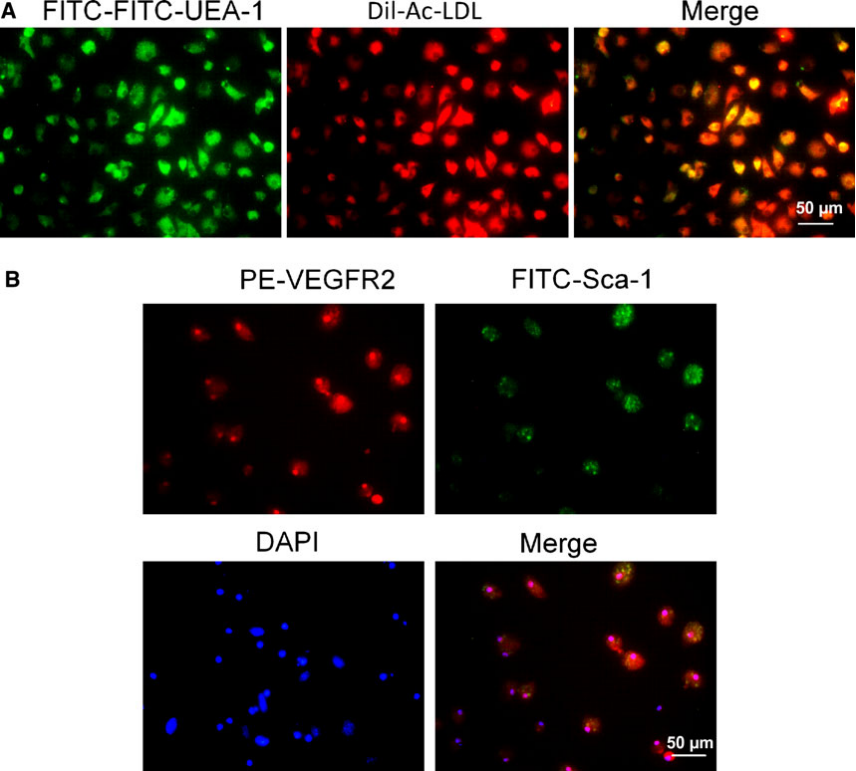
Characterization of bone marrow‐derived EPCs. (**A**) Dil‐ac‐LDL uptake and a FITC‐UEA‐1 binding assay showed that EPCs were Dil‐ac‐LDL and FITC‐UEA‐1 positive, respectively. (**B**) Immunofluorescence staining of cell surface markers VEGFR2 and Sca‐1 showed that EPCs were VEGFR2 and Sca‐1 double positive.

MNCs isolated from bone marrow were cultured in EGM‐2 medium on vitronectin‐coated culture dishes. By 14–21 days of culture, endothelium‐like cells with the cobblestone‐like morphology of late EPCs were nearly confluent. Late EPCs were further characterized by Dil‐ac‐LDL uptake and FITC‐UEA‐1 lectin binding. As shown in Figure 3A, most late EPCs were positive for Dil‐ac‐LDL and FITC‐UEA‐1 lectin staining. To further confirm the identity of these cells, immunofluorescence staining was performed to analyse the expression of specific cell markers (Fig. 3B, upper panel). The results showed that the majority of cells are both VEGFR2 and Sca‐1 positive (Fig. 3B, lower panel).

### Sitagliptin improves the survival and angiogenic function of EPCs treated with HG

**Figure 4:**
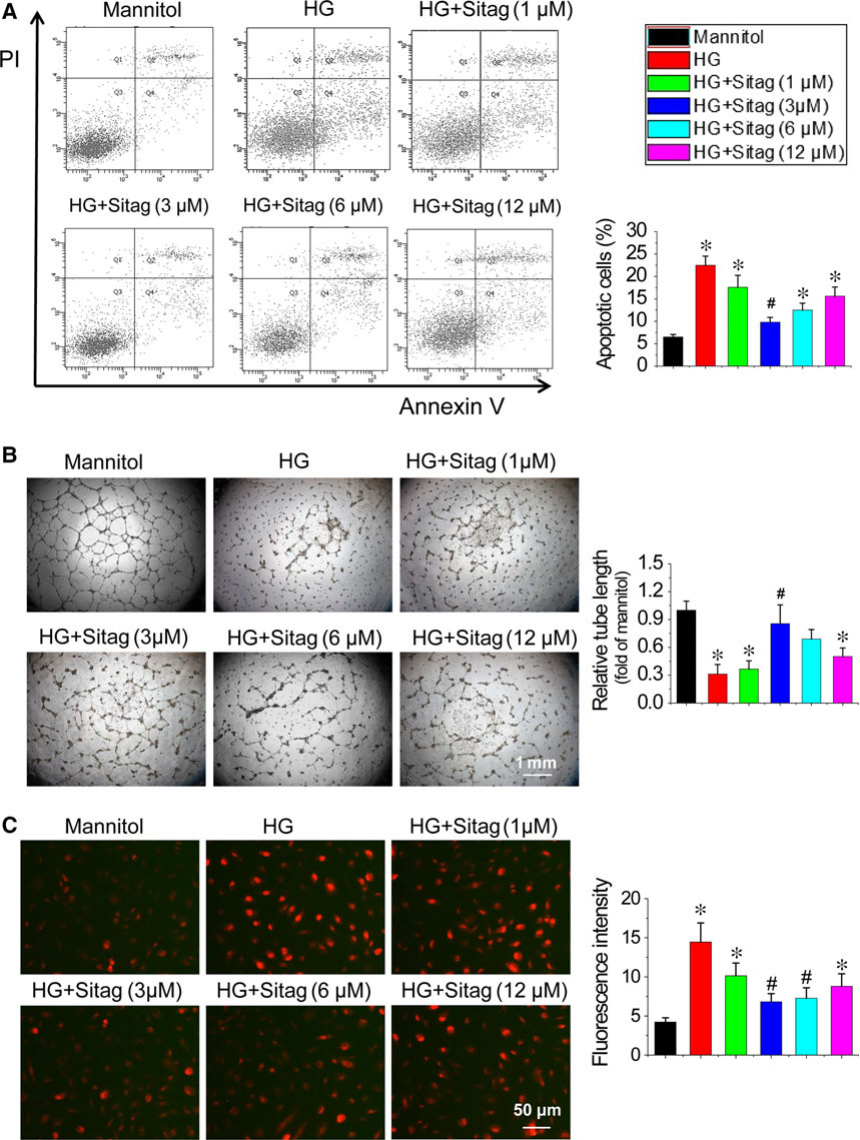
Sitagliptin improves the survival and angiogenic function of EPCs treated with high glucose (HG). EPCs were exposed to HG (25 mmol/l) with or without different doses of sitagliptin. (**A**) The apoptosis of EPCs was analysed by flow cytometry using Annexin V/propidium iodide (PI) staining after exposure to HG for 24 hrs. Apoptotic cells were defined as Annexin V^+^/PI ^‐^. (**B**) The effects of sitagliptin on the angiogenic function of EPCs after HG treatment for 12 hrs were determined by a tube formation assay. Tube length was normalized to the mannitol control group. (**C**) The anti‐oxidative effect of sitagliptin was determined by fluorescent probe DHE staining after exposure to HG for 6 hrs, and the fluorescence intensity of DHE was measured by a fluorescence microplate reader. Three independent experiments were performed for each study. Data shown in graphs represent the means ± S.D. *P < 0.05, vs mannitol control group; ^#^ *P* < 0.05, vs HG treatment group.

To validate the potency of sitagliptin, DPP‐4 activity was measured in the medium of EPCs using a DPP‐4 Activity Assay Kit. As expected, HG increased the DPP‐4 activity of EPCs, which was inhibited by sitagliptin treatment in a dose‐dependent manner (Figure S1). To determine whether DPP‐4 inhibition could restore the survival and angiogenic function of EPCs under diabetic conditions, EPCs were exposed to HG to mimic the dysmetabolic stress of hyperglycaemia. The results demonstrated that HG significantly increased EPC apoptosis, as measured by Annexin V/PI staining (Fig. 4A). Sitagliptin treatment dose dependently prevented HG‐induced apoptosis of EPCs within the lower dose range, although this protection was blunted at higher doses (Fig. 4A). Tube formation assays were used as one measure of angiogenic function *in vitro*. Similarly, HG significantly impaired the tube formation abilities of EPCs, and sitagliptin treatment significantly prevented the detrimental effects of HG on EPCs function at the same dose levels that prevented apoptosis (Fig. 4B).

### Sitagliptin attenuates ROS levels in EPCs treated with HG

Oxidative stress is a major factor responsible for the dysfunction of diabetic EPCs 19, 20. We used DHE staining to show superoxide production in EPCs. The results demonstrated that HG significantly increased superoxide production levels, whereas sitagliptin treatment attenuated HG‐induced superoxide production in EPCs at the same optimal dose level that prevented apoptosis and protected angiogenic function (Fig. 4C). Therefore, the optimal dose of sitagliptin at 3 μmol/l in EPCs protection was used in the following mechanistic studies.

### Sitagliptin improves the survival and function of EPCs treated with HG *via* augmenting autophagy

**Figure 5:**
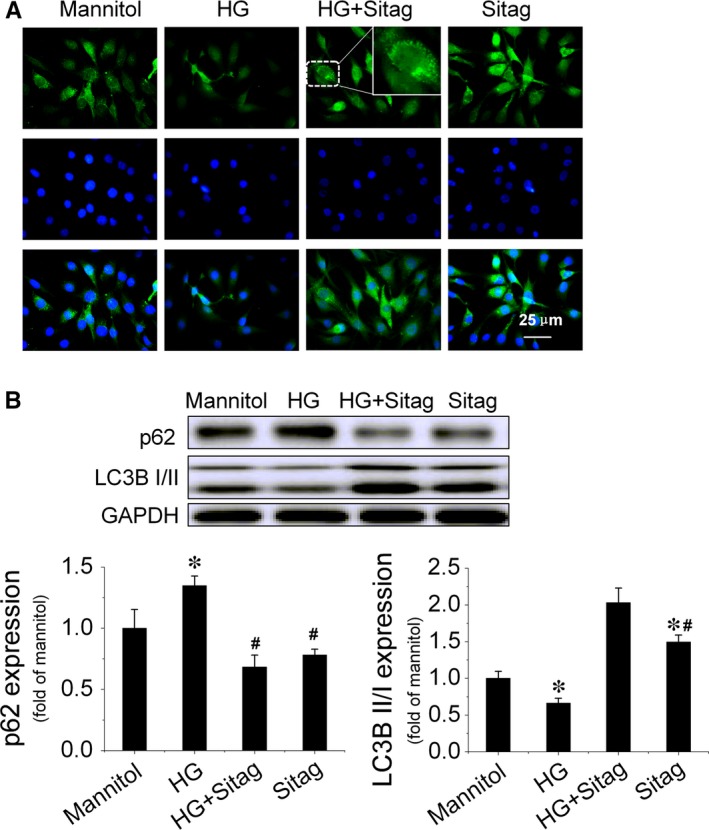
Sitagliptin augments autophagy in EPCs under HG treatment conditions. After treatment with HG in the presence or absence of sitagliptin (3 μmol/l) for 24 hrs, (**A**) the distribution of LC3 puncta was detected by immunofluorescence staining, and (**B**) the expression levels of LC3II/LC3I and p62 were detected by Western blotting. Three independent experiments were performed for each study. Data shown in graphs represent the means ± S.D. *P < 0.05, vs mannitol control group; ^#^ *P* < 0.05, vs HG treatment group.

Under diabetic conditions, impaired autophagy contributes to endothelial dysfunction, and restoration of autophagy can improve endothelial cell survival and function 21. In this study, we found that sitagliptin improved the survival and function of EPCs treated with HG. Therefore, we investigated whether the protective effects of sitagliptin on EPCs were mediated by autophagy activation in the following studies. EPCs were treated with HG (25 mmol/l) in the presence or absence of sitagliptin (3 μmol/l), and cells treated with 5.5 mmol/l glucose plus 20.5 mmol/l mannitol were used as a control. Immunofluorescence staining analysis demonstrated that elevated glucose levels reduced the number and distribution of LC3 puncta staining, suggesting a reduction in autophagosome formation, which was significantly prevented by sitagliptin treatment (Fig. 5A). In addition, we also detected the expression of p62 and the ratio of LC3II to LC3I by Western blotting. The results showed that the ratio of LC3II to LC3I decreased in EPCs with HG treatment, which was reversed by sitagliptin, whereas p62 expression exhibited the opposite pattern (Fig. 5B). These results indicate that sitagliptin activates autophagy in EPCs under HG treatment conditions.

**Figure 6:**
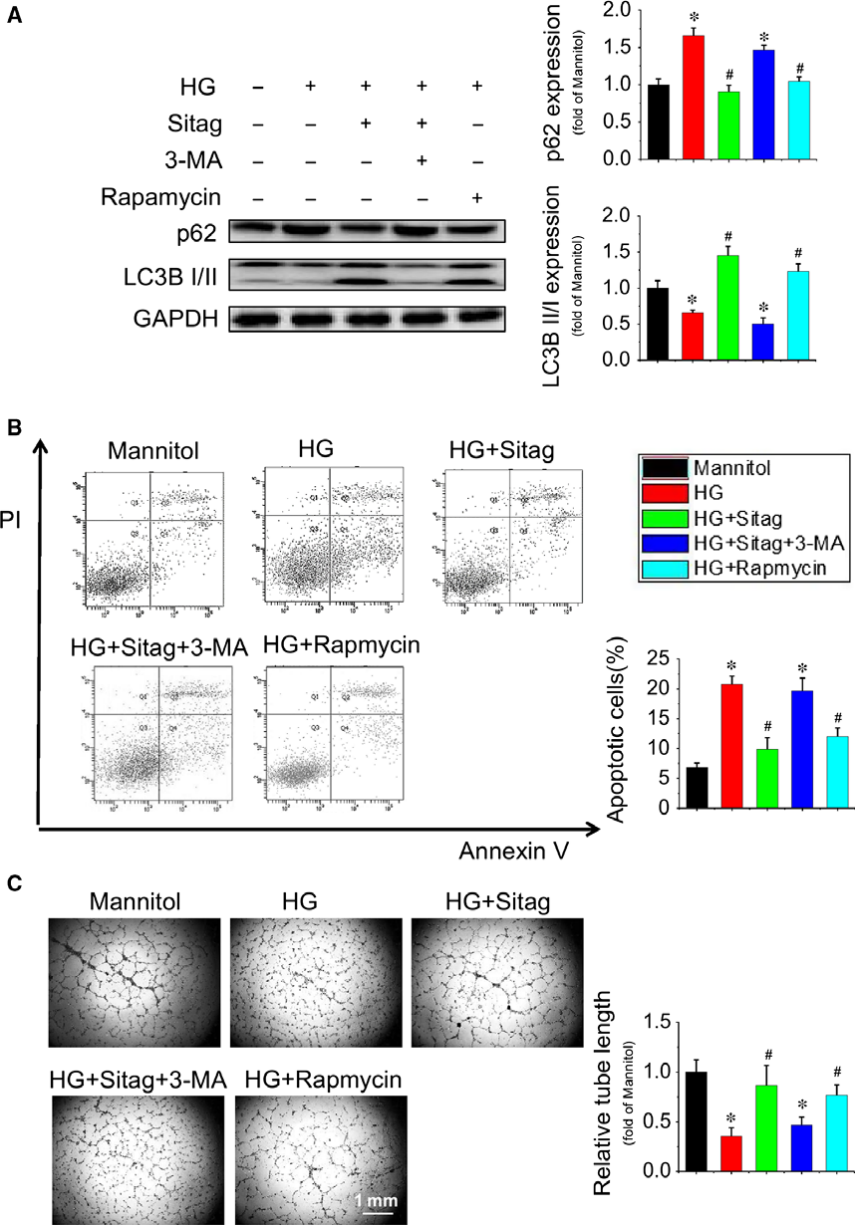
Sitagliptin improves the survival and function of EPCs treated with high glucose (HG) *via* augmenting autophagy. EPCs were pretreated with or without the autophagy inhibitor 3‐MA (5 mmol/l) or the autophagy activator rapamycin (10 μmol/l) for 30 min. and then exposed to HG in the presence or absence of sitagliptin (3 μmol/l). (**A**) The LC3 and p62 expression levels in EPCs were evaluated by Western blotting. (**B**) The apoptosis of EPCs was analysed by flow cytometry using Annexin V/PI staining. (**C**) The angiogenic function of EPCs was determined by a tube formation assay, and the tube length was normalized to the mannitol control group. Three independent experiments were performed for each study. Data shown in graphs represent the means ± S.D. *P < 0.05, vs mannitol control group; ^#^ *P* < 0.05, vs HG treatment group.

To confirm whether the protective effects of sitagliptin on EPCs were dependent on augmented autophagy, the autophagy inhibitor 3‐MA and the activator rapamycin were tested against the protection of sitagliptin. The result showed that inhibition of autophagy by 3‐MA completely abolished the sitagliptin‐stimulated increase in LC3II and decrease in p62 expression in EPCs (Fig. 6A), resulting in complete abolishment of sitagliptin‐induced cell survival (Fig. 6B) and tube formation (Fig. 6C) in EPCs under HG treatment conditions, whereas activation of autophagy by rapamycin almost completely prevented HG‐induced pathological changes in EPCs, which are comparable to that of sitagliptin treatment (Fig. 6A–C). These results suggest that sitagliptin preserves EPC function *via* restoring autophagy in EPCs.

### Sitagliptin restores autophagy in EPCs treated with HG *via* activating the AMPK/ULK1 signalling pathway

**Figure 7:**
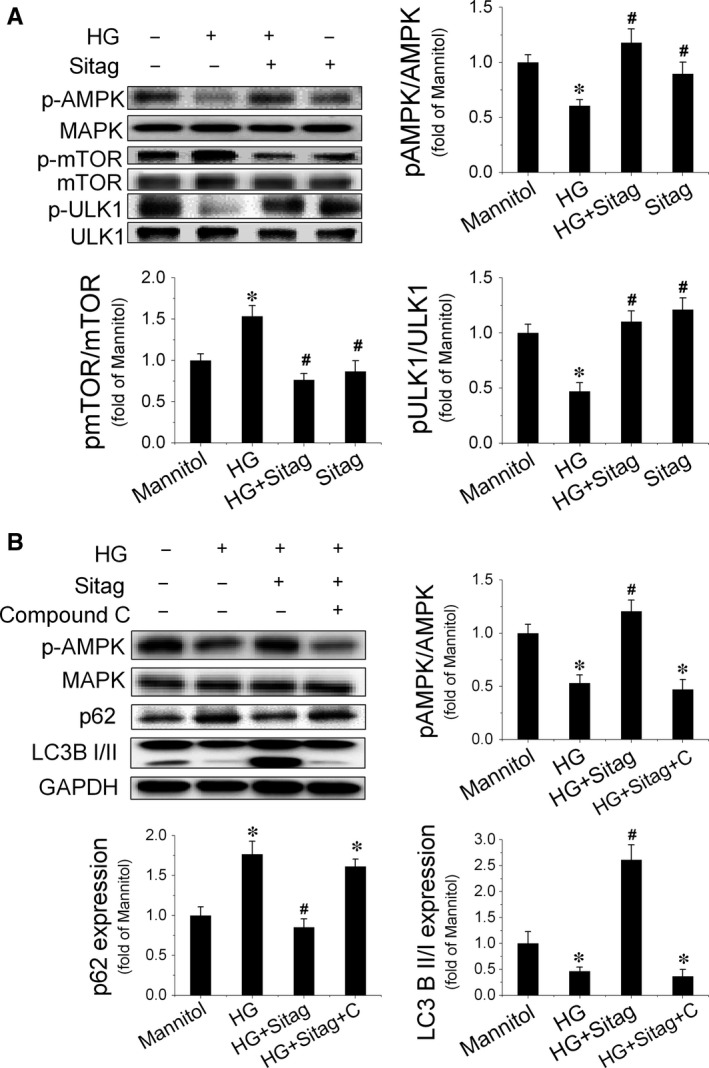
Sitagliptin restores autophagy in EPCs treated with high glucose (HG) *via* activating the AMPK/ULK signalling pathway. EPCs were pretreated with or without the AMPK inhibitor compound C (10 μmol/l) for 30 min. and then exposed to HG in the presence or absence of sitagliptin (3 μmol/l) for 24 hrs. (**A**) The phosphorylation of AMPK, mTOR and ULK1, and (**B**) the expression levels of p62 and LC3 were evaluated by Western blotting. Three independent experiments were performed for each study. Data shown in graphs represent the means ± S.D. *P < 0.05, vs mannitol control group; ^#^ *P* < 0.05, vs HG treatment group.

AMPK/ULK1 signalling is critical for autophagy regulation under diabetic conditions 22, 23. A previous study has shown that sitagliptin can activate AMPK 24. Therefore, we first detected AMPK activation in EPCs. The results showed that HG inhibited AMPK activity, as estimated by the decreased phosphorylation of AMPK, whereas sitagliptin treatment prevented HG‐induced decreases in the phosphorylation of AMPK (Fig. 7A). Meanwhile, we also found that sitagliptin prevented HG‐induced phosphorylation of mTOR, which was accompanied by an increase in the phosphorylation of ULK1 at Ser555 (Fig. 7A). To further confirm the critical role of AMPK activation in sitagliptin‐induced augmentation of autophagy in EPCs, the AMPK inhibitor compound C was tested against sitagliptin‐induced activation of autophagy. The result showed that compound C completely abolished the sitagliptin‐stimulated increase in LC3II and decrease in p62 expression in EPCs (Fig. 7B). These results suggest that AMPK is a critical mediator of the protective effects of sitagliptin on EPC function *via* augmenting autophagy.

## Discussion

The present study provides three new lines of evidence demonstrating the benefits of sitagliptin in ischaemic angiogenesis in type 2 diabetic mice *in vivo* and the angiogenic function of EPCs *in vitro*. The first novel finding is that sitagliptin treatment enhances blood perfusion and angiogenesis in HLI in *db/db* type 2 diabetic mice by increasing the circulating number of EPCs; the second innovative finding is that sitagliptin protects EPCs against diabetic stress conditions, which is mediated by restoring the autophagy of EPCs; and the third novel finding is that sitagliptin restores EPC autophagy, which is mediated by the AMPK/mTOR/ULK1 signalling pathway.

Previous studies have shown that DPP‐4 inhibitors can improve blood flow recovery and angiogenesis in critical limb ischaemia models, which are accompanied by an increase in circulating EPCs 8, 9. In the present study, for the first time, we revealed that sitagliptin treatment improves blood perfusion and enhances angiogenesis in HLI in *db/db* type 2 diabetic mice (Fig. 1), which is consistent with previous findings in normal mice 9, 25. We also found that sitagliptin treatment increased the number of circulating EPCs (Fig. 2D) and elevated the expression of the chemokine SDF‐1 in plasma (Fig. 2B). SDF‐1 is a substrate of DPP‐4, which proteolytically cleaves SDF‐1 and attenuates the interaction of SDF‐1 with its receptor CXCR4 26, 27. SDF‐1 is a critical factor in EPC mobilization from bone marrow to peripheral blood and homing to ischaemic sites *via* interacting with its receptor CXCR4 28. Therefore, sitagliptin‐induced enhancement of EPC mobilization and promotion of ischaemic blood perfusion and angiogenesis are most likely mediated by protecting SDF‐1 from DPP‐4 cleavage. In addition, our study demonstrated that sitagliptin also increased the proangiogenic factor SDF‐1 and VEGF expression in ischaemic tissue (Fig. 2C), indicating that sitagliptin‐augmented ischaemic angiogenesis may also attribute to a proangiogenic factor‐mediated paracrine mechanism.

Accumulating studies have demonstrated that the beneficial effects of DPP‐4 inhibition on vascular damage are mainly mediated by mobilization of EPCs 7, 8, 25; however, the direct effects of DPP‐4 inhibition on EPCs biological function have been largely neglected, especially under diabetic conditions. In a previous study, researchers have presented preliminary data showing the direct effects of the DPP‐4 inhibitors sitagliptin and vildagliptin on human EPCs under basal conditions 29. The authors found that DPP‐4 inhibition prevented the spontaneous apoptosis, enhanced cell proliferation and the expression levels of VEGF, VEGFR‐2 and eNOS in EPCs, which were accompanied by SDF‐1/CXCR4 signalling activation, and blockade of the SDF‐1/CXCR4 signalling pathway by AMD3100 resulted in increased apoptosis and the inhibition of cell proliferation and the expression levels of VEGF, VEGFR‐2 and eNOS in EPCs 29. In the present study, we further evaluated the direct effects of sitagliptin on EPC survival and angiogenic function under diabetic conditions for the first time (Fig. 4). We found that direct incubation of EPCs with sitagliptin significantly attenuated HG‐induced apoptosis and angiogenic dysfunction (Fig. 4A and B), which is consistent with previous findings that direct incubation of mesenchymal stem cells with sitagliptin remarkably attenuates hypoxia‐induced apoptosis^30^. More recently, Pujadas *et al*. 31 found that teneligliptin, another DPP‐4 inhibitor, reduced pro‐apoptotic gene expression levels and ameliorated oxidative stress in human umbilical vein endothelial cells under HG conditions. In the present study, we also found that sitagliptin attenuated the ROS accumulation in EPCs induced by HG (Fig. 4C). These results indicate that sitagliptin‐mediated improvement of the survival and angiogenic function of EPCs may attribute to sitagliptin‐induced attenuation of ROS production and accumulation under HG conditions.

Autophagy plays an important role in cellular homeostasis through the degradation and recycling of organelles, such as mitochondria or the endoplasmic reticulum, that are closely related to the pathogenesis of diabetes 32, 33. Autophagy can be induced by conditions such as starvation or inflammation but can also occur constitutively under non‐starvation conditions in basal autophagy, a process critical for the maintenance of cellular homoeostasis in the vasculature 33. The level of autophagy in EPCs from diabetic patients is decreased, and upregulating autophagy can improve the survival and function of EPCs under diabetic conditions^34^. Recently, the DPP‐4 inhibitor vildagliptin has been reported to reduce acute mortality after myocardial infarction with the restoration of autophagy in type 2 diabetes 11. In the present study, we found that HG treatment decreased the autophagy level in EPCs and that sitagliptin could restore the autophagy levels (Fig. 5A and B). Furthermore, the protective effects of sitagliptin on EPCs are orchestrated by the induction of autophagy as the autophagy inhibitor, 3‐MA, abrogated sitagliptin‐induced protection against apoptosis and angiogenic dysfunction under HG conditions (Fig. 6).

However, the role of ROS in the induction of autophagy still remains controversial. For example, recent studies have found that ROS inhibits autophagy 35, 36, whereas other studies have shown that natural compounds can protect cells against ROS damage *via* elevating autophagy 37, 38. In our study, we found that sitagliptin induced the autophagy of EPCs under HG conditions, which was accompanied by reduced ROS levels, indicating a possible link between sitagliptin‐induced autophagy and the reduced ROS levels of EPCs. The underlying mechanism needs to be investigated in future studies.

AMPK is a serine/threonine kinase that stimulates catabolic processes and inhibits anabolic processes to restore ATP levels when cellular energy is low 39. It is well‐known that activation of AMPK promotes starvation‐induced autophagy through interactions with mTORC1 and/or ULK‐1 40, 41. However, whether AMPK regulates basal autophagy in the excess nutrient environment is controversial. He *et al*. 42 found that activation of AMPK overcomes the autophagy impairment in myoblasts induced by HG; in addition, the authors also found that activation of AMPK restores autophagy in rodent macrophages exposed to palmitate, following stimulation with lipopolysaccharide 43. However, in human aortic endothelial cells, AMPK activation cannot restore autophagy impaired by HG and palmitate 44. In the present study, we showed that sitagliptin preserved the phosphorylation of AMPK and ULK1 and inhibited the phosphorylation of mTOR under HG treatment conditions, eventually resulting in the restoration of autophagy in EPCs, which could be blocked by the AMPK inhibitor compound C (Fig. 7). These findings indicate that sitagliptin‐induced restoration of autophagy in EPCs under HG conditions is mediated by the activation of the AMPK signalling pathway. However, how sitagliptin activates AMPK‐mediated autophagy signalling in EPCs under diabetic conditions warrants further investigation.

## Conflict of interest

The authors confirm that there are no conflicts of interest.

## Supporting information


**Figure S1** Sitagliptin significantly decreased the DPP‐4 activity of EPCs treated with HG.

## References

[1] UK Prospective Diabetes Study (UKPDS) . VIII. Study design, progress and performance. Diabetologia. 1991; 34: 877–90. 1778353

[2] Rahman S , Rahman T , Ismail AA , *et al* Diabetes‐associated macrovasculopathy: pathophysiology and pathogenesis. Diabetes Obes Metab. 2007; 9: 767–80. 17924861 10.1111/j.1463-1326.2006.00655.x

[3] Meijer AJ , Codogno P . Autophagy: a sweet process in diabetes. Cell Metab. 2008; 8: 275–6. 18840355 10.1016/j.cmet.2008.09.001

[4] Georgescu A . Vascular dysfunction in diabetes: the endothelial progenitor cells as new therapeutic strategy. World J Diabetes. 2011; 2: 92–7. 21860692 10.4239/wjd.v2.i6.92 PMC3158877

[5] Herrera C , Morimoto C , Blanco J , *et al* Comodulation of CXCR4 and CD26 in human lymphocytes. J Biol Chem. 2001; 276: 19532–9. 11278278 10.1074/jbc.M004586200

[6] Deng X , Szabo S , Chen L , *et al* New cell therapy using bone marrow‐derived stem cells/endothelial progenitor cells to accelerate neovascularization in healing of experimental ulcerative colitis. Curr Pharm Des. 2011; 17: 1643–51. 21548863 10.2174/138161211796197007

[7] Aso Y , Jojima T , Iijima T , *et al* Sitagliptin, a dipeptidyl peptidase‐4 inhibitor, increases the number of circulating CD34(+)CXCR4(+) cells in patients with type 2 diabetes. Endocrine. 2015; 50: 659–64. 26209038 10.1007/s12020-015-0688-5

[8] Chua S , Sheu JJ , Chen YL , *et al* Sitagliptin therapy enhances the number of circulating angiogenic cells and angiogenesis‐evaluations in vitro and in the rat critical limb ischemia model. Cytotherapy. 2013; 15: 1148–63. 23849976 10.1016/j.jcyt.2013.05.005

[9] Shih CM , Chen YH , Lin YW , *et al* MK‐0626, a dipeptidyl peptidase‐4 inhibitor, improves neovascularization by increasing both the number of circulating endothelial progenitor cells and endothelial nitric oxide synthetase expression. Curr Med Chem. 2014; 21: 2012–22. 24059225 10.2174/09298673113206660273

[10] Ku HC , Chen WP , Su MJ . DPP4 deficiency preserves cardiac function *via* GLP‐1 signaling in rats subjected to myocardial ischemia/reperfusion. Naunyn‐Schmiedeberg's Arch Pharmacol. 2011; 384: 197–207. 21748358 10.1007/s00210-011-0665-3

[11] Murase H , Kuno A , Miki T , *et al* Inhibition of DPP‐4 reduces acute mortality after myocardial infarction with restoration of autophagic response in type 2 diabetic rats. Cardiovasc Diabetol. 2015; 14: 103. 26259714 10.1186/s12933-015-0264-6 PMC4531441

[12] Tan Y , Li Y , Xiao J , *et al* A novel CXCR4 antagonist derived from human SDF‐1beta enhances angiogenesis in ischaemic mice. Cardiovasc Res. 2009; 82: 513–21. 19196827 10.1093/cvr/cvp044 PMC2682612

[13] Asahara T , Murohara T , Sullivan A , *et al* Isolation of putative progenitor endothelial cells for angiogenesis. Science (New York, NY). 1997; 275: 964–7. 10.1126/science.275.5302.964 9020076

[14] Jung SY , Choi JH , Kwon SM , *et al* Decursin inhibits vasculogenesis in early tumor progression by suppression of endothelial progenitor cell differentiation and function. J Cell Biochem. 2012; 113: 1478–87. 22298358 10.1002/jcb.24085

[15] Dai X , Tan Y , Cai S , *et al* The role of CXCR7 on the adhesion, proliferation and angiogenesis of endothelial progenitor cells. J Cell Mol Med. 2011; 15: 1299–309. 21418513 10.1111/j.1582-4934.2011.01301.x PMC4373330

[16] Steinmetz M , Lucanus E , Zimmer S , *et al* Mobilization of sca1/flk‐1 positive endothelial progenitor cells declines in apolipoprotein E‐deficient mice with a high‐fat diet. J Cardiol. 2015; 66: 532–8. 25818640 10.1016/j.jjcc.2015.02.008

[17] Haberzettl P , Lee J , Duggineni D , *et al* Exposure to ambient air fine particulate matter prevents VEGF‐induced mobilization of endothelial progenitor cells from the bone marrow. Environ Health Perspect. 2012; 120: 848–56. 22418586 10.1289/ehp.1104206 PMC3385427

[18] Dai X , Yan X , Zeng J , *et al* Elevating CXCR7 improves angiogenic function of EPCs *via* Akt/GSK‐3beta/Fyn‐mediated Nrf2 activation in diabetic limb ischemia. Circ Res. 2017; 120: e7–23. 28137917 10.1161/CIRCRESAHA.117.310619 PMC5336396

[19] Dernbach E , Urbich C , Brandes RP , *et al* Antioxidative stress‐associated genes in circulating progenitor cells: evidence for enhanced resistance against oxidative stress. Blood. 2004; 104: 3591–7. 15161665 10.1182/blood-2003-12-4103

[20] Sorrentino SA , Bahlmann FH , Besler C , *et al* Oxidant stress impairs in vivo reendothelialization capacity of endothelial progenitor cells from patients with type 2 diabetes mellitus: restoration by the peroxisome proliferator‐activated receptor‐gamma agonist rosiglitazone. Circulation. 2007; 116: 163–73. 17592079 10.1161/CIRCULATIONAHA.106.684381

[21] Fetterman JL , Holbrook M , Flint N , *et al* Restoration of autophagy in endothelial cells from patients with diabetes mellitus improves nitric oxide signaling. Atherosclerosis. 2016; 247: 207–17. 26926601 10.1016/j.atherosclerosis.2016.01.043 PMC4913892

[22] Chang C , Su H , Zhang D , *et al* AMPK‐dependent phosphorylation of GAPDH triggers Sirt1 activation and is necessary for autophagy upon glucose starvation. Mol Cell. 2015; 60: 930–40. 26626483 10.1016/j.molcel.2015.10.037

[23] Kim J , Kundu M , Viollet B , *et al* AMPK and mTOR regulate autophagy through direct phosphorylation of Ulk1. Nat Cell Biol. 2011; 13: 132–41. 21258367 10.1038/ncb2152 PMC3987946

[24] Zeng Y , Li C , Guan M , *et al* The DPP‐4 inhibitor sitagliptin attenuates the progress of atherosclerosis in apolipoprotein‐E‐knockout mice *via* AMPK‐ and MAPK‐dependent mechanisms. Cardiovasc Diabetol. 2014; 13: 32. 24490809 10.1186/1475-2840-13-32 PMC3916068

[25] Huang CY , Shih CM , Tsao NW , *et al* Dipeptidyl peptidase‐4 inhibitor improves neovascularization by increasing circulating endothelial progenitor cells. Br J Pharmacol. 2012; 167: 1506–19. 22788747 10.1111/j.1476-5381.2012.02102.x PMC3514763

[26] Busso N , Wagtmann N , Herling C , *et al* Circulating CD26 is negatively associated with inflammation in human and experimental arthritis. Am J Pathol. 2005; 166: 433–42. 15681827 10.1016/S0002-9440(10)62266-3 PMC1602320

[27] Wang W , Choi BK , Li W , *et al* Quantification of intact and truncated stromal cell‐derived factor‐1alpha in circulation by immunoaffinity enrichment and tandem mass spectrometry. J Am Soc Mass Spectrom. 2014; 25: 614–25. 24500701 10.1007/s13361-013-0822-7

[28] Wils J , Favre J , Bellien J . Modulating putative endothelial progenitor cells for the treatment of endothelial dysfunction and cardiovascular complications in diabetes. Pharmacol Ther. 2017; 170: 98–115. 27773788 10.1016/j.pharmthera.2016.10.014

[29] Liu F , Huang G , JZ T , *et al* DPP4 inhibitors promote biological functions of human endothelial progenitor cells by targeting the SDF‐1/CXCR4 signaling pathway. Arch Biol Sci. 2016; 68: 207–16.

[30] Wang XM , Yang YJ , Wu YJ , *et al* Attenuating hypoxia‐induced apoptosis and autophagy of mesenchymal stem cells: the potential of sitagliptin in stem cell‐based therapy. Cell Physiol Biochem. 2015; 37: 1914–26. 26584290 10.1159/000438552

[31] Pujadas G , De Nigris V , Prattichizzo F , *et al* The dipeptidyl peptidase‐4 (DPP‐4) inhibitor teneligliptin functions as antioxidant on human endothelial cells exposed to chronic hyperglycemia and metabolic high‐glucose memory. Endocrine. 2017; 56: 509–20. 27530507 10.1007/s12020-016-1052-0 PMC5435779

[32] Levine B , Kroemer G . Autophagy in the pathogenesis of disease. Cell. 2008; 132: 27–42. 18191218 10.1016/j.cell.2007.12.018 PMC2696814

[33] Mizushima N . Autophagy in protein and organelle turnover. Cold Spring Harb Symp Quant Biol. 2011; 76: 397–402. 21813637 10.1101/sqb.2011.76.011023

[34] Chen W , Wu Y , Li L , *et al* Adenosine accelerates the healing of diabetic ischemic ulcers by improving autophagy of endothelial progenitor cells grown on a biomaterial. Sci Rep. 2015; 5: 11594. 26108983 10.1038/srep11594 PMC4479873

[35] Ci Y , Shi K , An J , *et al* ROS inhibit autophagy by downregulating ULK1 mediated by the phosphorylation of p53 in selenite‐treated NB4 cells. Cell Death Dis. 2014; 5: e1542. 25429619 10.1038/cddis.2014.506 PMC4260759

[36] Wang Q , Guo W , Hao B , *et al* Mechanistic study of TRPM2‐Ca(2 + )‐CAMK2‐BECN1 signaling in oxidative stress‐induced autophagy inhibition. Autophagy. 2016; 12: 1340–54. 27245989 10.1080/15548627.2016.1187365 PMC4968236

[37] Zhang S , Guo C , Chen Z , *et al* Vitexin alleviates ox‐LDL‐mediated endothelial injury by inducing autophagy *via* AMPK signaling activation. Mol Immunol. 2017; 85: 214–21. 28288411 10.1016/j.molimm.2017.02.020

[38] Guo H , Chen Y , Liao L , *et al* Resveratrol protects HUVECs from oxidized‐LDL induced oxidative damage by autophagy upregulation *via* the AMPK/SIRT1 pathway. Cardiovasc Drugs Ther. 2013; 27: 189–98. 23358928 10.1007/s10557-013-6442-4

[39] Srivastava RA , Pinkosky SL , Filippov S , *et al* AMP‐activated protein kinase: an emerging drug target to regulate imbalances in lipid and carbohydrate metabolism to treat cardio‐metabolic diseases. J Lipid Res. 2012; 53: 2490–514. 22798688 10.1194/jlr.R025882 PMC3494254

[40] Liao LZ , Chen YL , Lu LH , *et al* Polysaccharide from Fuzi likely protects against starvation‐induced cytotoxicity in H9c2 cells by increasing autophagy through activation of the AMPK/mTOR pathway. Am J Chin Med. 2013; 41: 353–67. 23548125 10.1142/S0192415X13500262

[41] Li L , Chen Y , Gibson SB . Starvation‐induced autophagy is regulated by mitochondrial reactive oxygen species leading to AMPK activation. Cell Signal. 2013; 25: 50–65. 23000343 10.1016/j.cellsig.2012.09.020

[42] He C , Zhu H , Li H , *et al* Dissociation of Bcl‐2‐Beclin1 complex by activated AMPK enhances cardiac autophagy and protects against cardiomyocyte apoptosis in diabetes. Diabetes. 2013; 62: 1270–81. 23223177 10.2337/db12-0533 PMC3609561

[43] Wen H , Gris D , Lei Y , *et al* Fatty acid‐induced NLRP3‐ASC inflammasome activation interferes with insulin signaling. Nat Immunol. 2011; 12: 408–15. 21478880 10.1038/ni.2022 PMC4090391

[44] Weikel KA , Cacicedo JM , Ruderman NB , *et al* Glucose and palmitate uncouple AMPK from autophagy in human aortic endothelial cells. Am J Physiol Cell Physiol. 2015; 308: C249–63. 25354528 10.1152/ajpcell.00265.2014 PMC4312840

